# Interfacial Rheology of Surfactant–Asphaltene Systems: State of the Art and Implications for Enhanced Oil Recovery

**DOI:** 10.3390/ma18215036

**Published:** 2025-11-05

**Authors:** Maria Isabel Sandoval Martinez, Ronald Mercado, Arlex Chaves-Guerrero, Hassan Hassanzadeh

**Affiliations:** 1 Grupo de Investigación en Recobro Mejorado—GRM, Universidad Industrial de Santander, Bucaramanga 680002, Colombia; grm@uis.edu.co; 2 Grupo de Investigación en Fenómenos Interfaciales, Reología y Simulación de Transporte—FIRST, Universidad Industrial de Santander, Bucaramanga 680002, Colombia; achavesg@uis.edu.co; 3 Department of Chemical & Petroleum Engineering, Schulich School of Engineering, University of Calgary, Calgary, AB T2N 1N4, Canada; hhassanz@ucalgary.ca

**Keywords:** interfacial rheology, enhanced oil recovery, surfactants, asphaltenes, elasticity

## Abstract

The study of the viscoelastic properties of surfactants in Enhanced Oil Recovery (EOR) has gained significant attention due to the role of interface elasticity in improving oil recovery. Interfacial rheology has been demonstrated to be a valuable tool for designing more efficient surfactant formulations in different industries. This review summarizes the principles and methods used to understand interfacial rheology and its impact on oil recovery. The paper explores key processes, interactions, and parameters that influence the formation of viscous or elastic films in the presence of active components in petroleum systems. The main findings highlight the importance of achieving optimal rigidity and viscoelastic properties at the interface, which promotes the formation of continuous phase threads that can be more easily swept. The review emphasizes the significance of understanding intermolecular interactions between surfactants and asphaltenes, as well as the impact of surfactant concentration on the formation of more viscous or elastic interfaces. Despite the valuable insights provided by interfacial rheology, further research is required to refine surfactant-based EOR strategies to enhance petroleum processing and recovery.

## 1. Introduction

The selection of enhanced oil recovery (EOR) methods should be based on understanding the main forces governing oil entrapment in the porous medium, whether they are viscous or capillary forces [1]. Surfactant solutions are among the most widely used chemical-enhanced methods for residual oil recovery, as they can modify the water/oil interface properties and influence rock–fluid interactions, thereby reducing capillary forces [2]. In practice, surfactant formulations used in the petroleum industry include anionic, cationic, and nonionic types, each selected based on the reservoir conditions such as salinity, temperature, and rock wettability [3]. These compounds are designed to reduce interfacial tension, alter wettability, and stabilize or destabilize emulsions depending on the recovery strategy [4,5].

Most studies have focused on lowering interfacial tension below 10^−2^ mN/m to achieve capillary numbers greater than 10^−2^. However, these measurements are often conducted under steady-state conditions, which are unrealistic in the dynamic reservoir environment. This limitation arises from neglecting the continuous disturbances that the system experiences when surfactants are injected into the porous medium [6]. Moreover, surfactants can alter interfacial viscoelasticity, either increasing or decreasing it, which in turn affects the amount of oil trapped in the porous medium [7]. In other words, interfacial tension is not the only property influencing oil recovery during surfactant injection [8]. For instance, some studies have shown that formulations designed to reduce interfacial tension to the lowest value did not achieve the highest oil recovery factor [7,9,10]. This trend has been attributed to the interfacial viscoelastic properties of these systems. The key to optimal recovery lies in finding the right balance between interfacial viscoelasticity and tension, since systems with the lowest values of both properties tend to produce less oil. Conversely, systems with appropriate interfacial viscoelasticity help reduce snap-off and increase the coalescence speed of oil droplets during waterflooding, resulting in improved oil recovery [7,11].

Despite the points mentioned above, the effect of interfacial viscoelasticity on oil recovery performance is often overlooked in the design of surfactant formulations. This is due to the uncertainty surrounding this property when amphiphilic molecules are introduced [12,13]. Such a lack of understanding may limit the search for truly optimal formulations, often focusing solely on reducing interfacial tension, leading to the dismissal of formulations with lower surfactant concentrations. Therefore, this review aims to evaluate the approaches in the literature for assessing the effect of interfacial viscoelasticity during surfactant injection and to explain the interactions that influence this property, ultimately affecting the performance of enhanced oil recovery (EOR) technologies. The review is organized into eight sections. Section 1 introduces the principles and methods used for interfacial rheology measurements. Subsequent sections describe the snap-off mechanism as a key process of oil trapping in porous media and examine the role of surfactants in mobilizing oil trapped under such conditions. The later sections analyze the main processes, interactions, and parameters governing the formation of viscous or elastic interfacial films, with emphasis on surfactant–asphaltene systems. Finally, the review concludes with a discussion of future research directions and prospects derived from the insights presented.

## 2. Interfacial Rheology: Principles and Methods

Interfacial rheology has been defined as the science that studies the response of mobile interphases to deformation [14]. This is one of the most powerful tools for observing occurrences at the interface. This scientific discipline primarily examines three key areas: the dynamic interactions at the boundary between two fluids, how the interface responds to deformation, and the measurable impact that interface stresses exert on the hydrodynamics and rheology of the system’s constituent phases.

Interfacial rheology characterizes the flow and deformation behavior at the boundary between two immiscible fluids under applied stress or strain, using principles analogous to those of bulk rheology to assess interfacial viscosity and viscoelasticity. To study the behavior and strength of the interfacial layer, various interfacial rheology techniques are employed to determine the viscous and elastic coefficients. These techniques are generally divided into two primary categories: dilatational and shear methods.

### 2.1. Interfacial Shear Rheology Method

**Figure 1:**
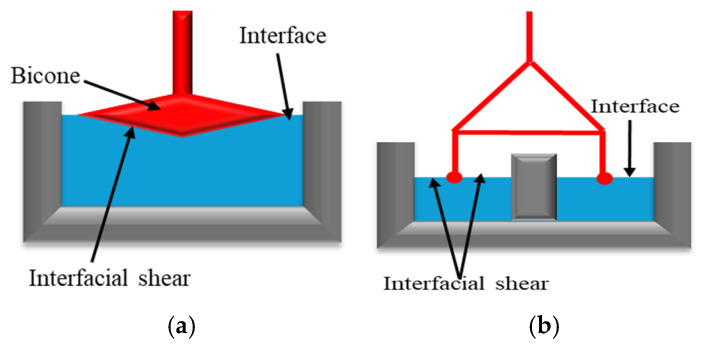
Interfacial Geometries examples: (**a**) Bicone type, adapted from Omari et al. [15]. (**b**) Double-wall ring adapted from Omari et al. [16].

This method involves interface deformation (modifying the shape without altering its area) by moving an object with variable geometry (needle, bi-cone, ring, Du Noüy ring, etc.; Figure 1) or by applying periodic oscillations. The resistance at the interface is measured by the rheometer’s sensor. The force or torque applied to the interface is used to estimate the interfacial stress. The object movement analysis enables the estimation of interfacial de-formation and its rate [15,16]. Using this information, the elastic (G′) and viscous (G″) components of shear viscosity can be determined.

The bicone and double-wall ring geometries are among the most used systems for interfacial rheology measurements. The bicone system (Figure 1a) provides a cost-effective, straightforward method for measuring shear stress at an interface [17]. However, it is primarily suited for stiff interfaces due to its sensitivity to relatively large shear stresses. As a result, this geometry is not ideal for characterizing fragile interfaces with very low interfacial viscosity. Additionally, its application is limited in systems with viscous sub-phases, as the drag forces exerted on the bicone can sometimes become non-negligible [15,18]. On the other hand, the double-wall ring (Figure 1b) features a lightweight geometry with a low moment of inertia, making it suitable for measuring interfacial properties at both viscous and viscoelastic interfaces in continuous-shear and oscillatory experiments [16,17,19]. However, its main drawback is the ring’s fragility, which may limit its durability and handling [15].

Interfacial shear rheology has traditionally been more popular among experimentalists. However, in recent years, researchers have raised concerns about the reproducibility of interfacial shear rheological data, which is often affected by the measurement technique and the system under study [20]. Additionally, this method poses challenges in accurately positioning the solid measuring geometry at the liquid–liquid interface, which can compromise measurement precision [21].

### 2.2. Dilatational Interfacial Rheology Method

**Figure 2:**
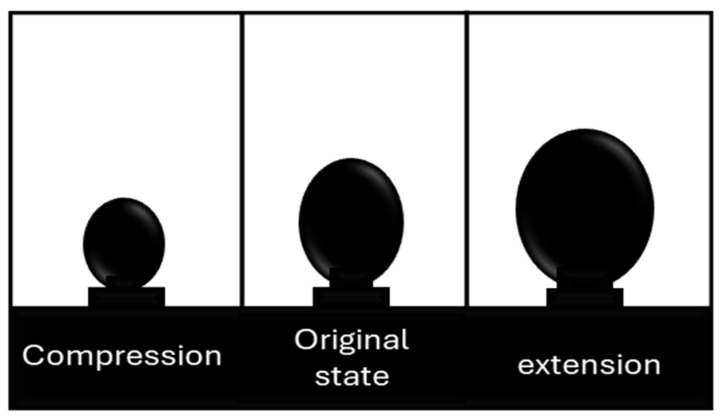
Differentially oscillating drop area. Adapted from Omari et al. [15].

Dilatational methods involve changing the surface area of the interface through periodic compression and expansion strains. As the surface area oscillates, gradients in interfacial tension develop due to the movement of molecules toward or away from each other [15,22]. A common technique to measure dilatational interfacial rheology is the oscillating pendant drop method. In this method, a droplet suspended at the tip of a needle is subjected to periodic strain by oscillating the drop’s surface area (Figure 2). The periodic stress response is then measured using pendant-drop tensiometry and axisymmetric drop-shape analysis [23].

**Figure 3:**
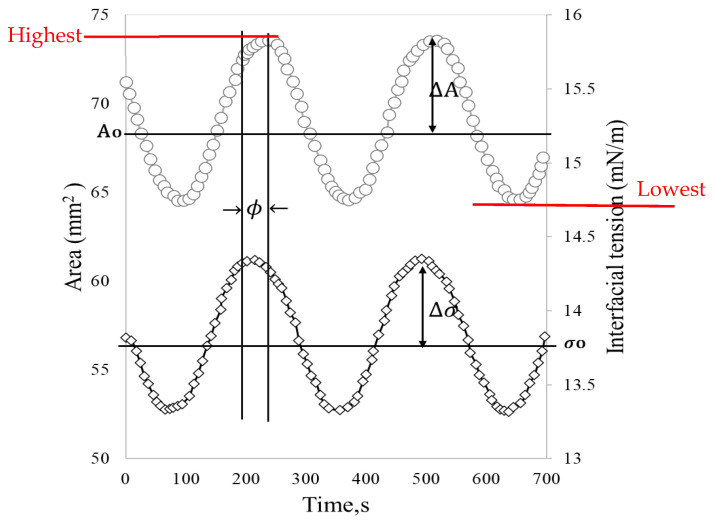
Stress response (interfacial stress) to oscillatory strain (surface area). Adapted from Freer et al. [23].

These oscillatory movements of expansion and contraction of the drop at a given frequency (ω) cause interfacial tension changes with sinusoidal behavior (Figure 3). For merely elastic interfaces, the dynamic interfacial tension response (γ(t)) immediately follows the area change (A(t)) without phase lag. However, in viscoelastic interfaces, most interfacial layers present a phase shift (ϕ) [24].

In the described interfacial rheology experiments, it is essential to ensure that the droplet remains in mechanical equilibrium [25], and the measurements are conducted within the linear viscoelastic region, defined as the strain range where stress and deformation maintain a linear relationship, thereby preventing damage to the interface during each measurement [6,26]. The critical amplitudes (change in area) and frequencies of the measurements can be determined through a sweep of these parameters, with values ranging between 2–10% and 0.01–0.1 Hz, respectively, to obtain reliable results [27,28]

Dynamic interfacial tension (σ) and the surface area (A) obtained through the drop shape analysis (DSA) are converted into a complex interfacial dilatational modulus (E*) through Equation (1).

(1) $$E^{*}=Ao\;\frac{d\sigma }{dA}=\left|Ao\;\frac{∆\sigma }{∆A}\right|$$

where Ao is the drop area in the original state, ∆σ and ∆A  are the difference between the highest and the lowest interfacial tension and the area, respectively. The interfacial dilatational module is divided into real and imaginary components that correspond to the elastic (E′) and viscous (E″) dilatational moduli, respectively. These moduli are obtained by adjusting the experimental data to Equations (2) and (3) and are used to determine the viscoelastic behavior of the interface [24].

(2) $$E^{\prime }=\left|Ao\;\frac{∆\sigma }{∆A}\right|\;cos(\phi )$$

(3) $$E^{\prime \prime }=\left|Ao\;\frac{∆\sigma }{∆A}\right|\;sin(\phi )$$

Depending on the viscoelasticity, a response lag may occur due to deformation. The lag is referred to as the phase angle (ϕ), which is a measure of the elasticity of the interface. When the film is purely elastic ϕ= 0° and when it is purely viscous *ϕ* = 90° [29]. From Equation (4), it is observable that the ratio of the storage and loss modulus (*tan* ϕ) can be used as a measure of the ratio of the elastic to the viscous nature of the monolayer [30].

(4) $$tan\;\phi =\frac{E^{\prime \prime }}{E^{\prime }}$$

Likewise, the interfacial dilatational viscosity can be calculated with this information and Equation (5).

(5) $$\mu_{d}=Ao\;\frac{d\sigma }{dA/dt}$$

**Figure 4:**
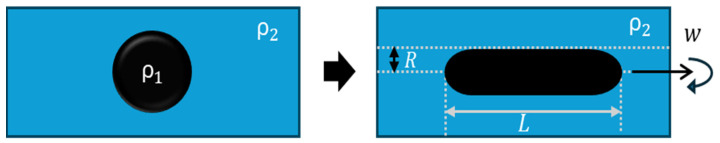
Schematic diagram of the spinning drop method. Modified from Omari et al. [15].

Another widely used technique is the spinning drop method, which operates on the same fundamental principle as the pendant drop method. Spinning drop tensiometry uses the optical contour analysis of a drop to determine the interfacial tension (Figure 4).

For interfacial rheology determination, the rotational velocity (ω) varies oscillatorily. In response to rotational velocity, the drop area is enlarged or reduced, and this information is used to estimate the interfacial tension for each value with Equation (6) [29].

(6) $$\sigma =\frac{∆\rho {w_{rot}}^{2}d^{3}}{32}$$

where d is the diameter of the droplet at the central equator in mm, Δ*ρ* is the density difference between the two fluids in g/mL, and $w_{rot}$ is the rotational velocity in rev/ms. The information obtained is similar to that depicted in Figure 3, and the calculation of the dilatational module and viscosity in the spinning drop is the same as was explained previously in Equations (1)–(6).

Table 1 displays the comparison between the above methods and the typical measurement parameters.

## 3. Oil Trapping by the Snap-Off Mechanism

During conventional waterflooding, a significant fraction of oil remains trapped in the porous medium due to capillary forces. Oil can be immobilized either as isolated droplets within individual pores, at the pore center, or attached to the wall, or as larger, interconnected patches spanning multiple pores surrounded by water [35,36]. Capillary trapping is primarily governed by two mechanisms: snap-off and bypass [37], with snap-off being the dominant contributor to residual oil saturation [38].

**Figure 5:**
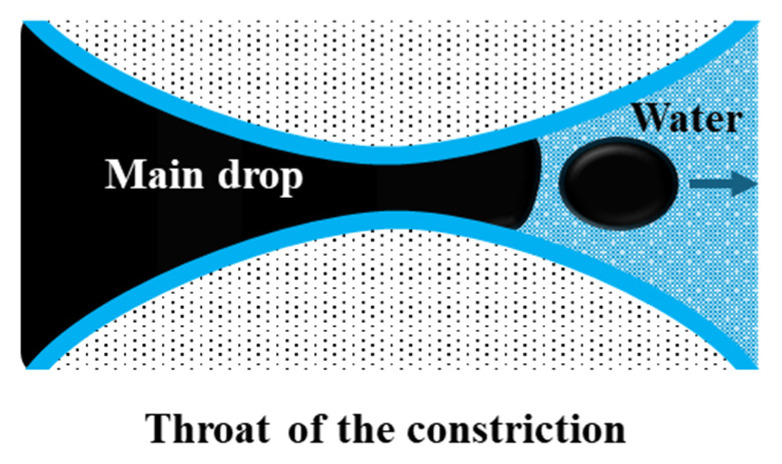
Representation of the snap-off phenomenon, adapted from Roof [41].

Snap-off is a capillary-driven instability that occurs primarily in water-wet systems during imbibition [39]. It occurs in pore constrictions characterized by a large body-to-throat ratio, resulting in a “bottle-neck” geometry (Figure 5). As water advances, it forms a wetting layer around the non-wetting oil phase. This layer thickens in the throat region, forcing elongated oil threads to neck, detach, and break into smaller droplets [37,40]. The process depends strongly on pore geometry, wettability, capillary number (the balance of viscous to interfacial forces), and the mobility ratio between the displacing and displaced phases [38,41].

At high oil saturations, droplet coalescence occurs at a rate comparable to snap-off, enabling the mobilization of oil banks [42]. As saturation decreases, however, oil ganglia fragment into disconnected droplets too small to reconnect, becoming irreversibly trapped in pores with high capillary pressures [1]. Under such conditions, conventional waterflooding is insufficient, and advanced recovery strategies are required.

Enhanced Oil Recovery (EOR) using chemical agents, particularly surfactants, has proven effective for mobilizing snap-off–trapped oil [43]. Surfactants migrate to the oil–water interface, reduce interfacial tension, and modify viscoelastic properties of the film. This dual effect not only lowers the energy barrier for droplet elongation and mobilization but also stabilizes elongated oil threads against premature breakup in pore constrictions [15]. By tuning both interfacial tension and interfacial rheology, surfactants enable the reconnection of disconnected oil ganglia into flowing banks and suppress the snap-off.

## 4. Impact of Surfactant Injection on the Oil Snap-Off Mechanism

The adsorption of surfactants at the oil–water interface reduces interfacial tension (IFT) and capillary forces, which promotes the elongation and mobilization of trapped oil droplets [44]. Lowering capillary pressure enables the injected fluid to penetrate smaller pores and displace residual oil, thereby reducing residual saturation and enhancing recovery efficiency. Conventional approaches suggest that IFT must be reduced to values near 10^−2^ mN/m to achieve significant mobilization [37,43]. However, the effect of surfactants on oil recovery cannot be explained solely by their capacity to lower IFT, since the viscoelastic properties of the interfacial film also play a decisive role.

Once surfactants are adsorbed, the interface behaves as a two-dimensional body with its own elasticity and viscosity [45]. These viscoelastic properties significantly influence the deformation and breakup of oil drops in pore throats. An interface with balanced viscoelasticity can suppress excessive droplet fragmentation during snap-off, reduce residual saturation, and improve oil recovery [46]. Interfacial rheology is closely related to emulsion stability, which is commonly explained by two complementary theories. According to surface tension theory, surfactants form viscoelastic films that reduce IFT and promote emulsification by dispersing residual oil into smaller, mobile droplets [47,48,49]. Detached oil droplets then adopt spherical or ellipsoidal shapes, forming pore-scale emulsions that are transported downstream [50]. Repulsion theory, on the other hand, emphasizes the role of viscoelastic films as elastic membranes that provide steric and electrostatic barriers against coalescence, thereby enhancing suspension stability [45,50]. While these effects are beneficial for mobilization within porous media, excessive emulsion stability can complicate surface separation processes [51]. For this reason, the design of surfactant formulations must carefully balance interfacial viscosity and elasticity to maximize displacement while avoiding operational issues.

Some studies have demonstrated that the lowest IFT does not always correspond to the highest oil recovery [7,9,10]. Instead, recovery depends on the interplay between IFT and viscoelasticity. Excessive viscoelasticity may hinder oil bank formation by suppressing droplet coalescence, whereas interfaces with moderate elasticity facilitate droplet reconnection and the formation of continuous oil threads, which are more effectively displaced [11,22]. For instance, García-Olvera et al. [11] evaluated the interfacial rheological behavior of different crude oils using a double-wall ring rheometer. They found that an interfacial phase angle of approximately 26°, which means the interface was more elastic than viscous, led to 81% oil recovery, even though the system did not exhibit the lowest interfacial tension (Table 2). Márquez et al. [22] further emphasized that optimal EOR systems must combine low rigidity, which promotes coalescence after snap-off, with an elastic modulus greater than the viscous component (E′ > E″, phase angle < 45°), ensuring that elongated oil threads resist breaking up into small droplets.

Moreover, the complexity of surfactant behavior is further increased by the active components of crude oil, particularly asphaltenes and resins. These polar species can ad-sorb at the interface via electrostatic interactions, thereby reducing IFT and forming rigid viscoelastic films [6,52]. Depending on the formulation, surfactants may compete with or cooperate with these components. Highly active surfactants may displace asphaltenes, resulting in weaker but lower interfacial tension (IFT) interfaces. In contrast, surfactants with intermediate interfacial activity and high hydrophobic character may promote cooperative interactions with asphaltenes, producing cross-linked clusters that enhance elasticity and rigidity [7,9]. These cooperative or competitive mechanisms illustrate how surfactant–asphaltene interactions strongly condition interfacial behavior and, consequently, oil recovery outcomes.

Altogether, the findings from experimental and theoretical studies highlight that the most favorable conditions for oil recovery occur in an intermediate viscoelastic regime. Interfaces must be elastic enough to sustain elongated oil threads but not so rigid as to prevent coalescence. Phase angles between 0° and 45° have been identified as favorable, with ~26° reported as optimal for achieving higher oil displacement efficiency in the laboratory by Garcia-Olvera et al. [11].

Interfacial rheology, therefore, represents a valuable complement to IFT measurements in the rational design of surfactant formulations for EOR, as it captures the dynamic response of the oil–brine interface under continuous disturbance and provides insight into snap-off suppression and oil mobilization [7,8,11]. Nevertheless, systematic dilatational rheology studies in real EOR systems remain limited, reflecting the experimental challenges of handling complex surfactant–crude oil mixtures [13]. In the following section, we analyze the processes and conditions by which surfactant systems in oil–water environments generate viscous or elastic interfacial films.

## 5. Formation and Organization of Interfaces

The type of film formed at the oil–water interface upon the addition of amphiphilic molecules depends on the interplay among diffusion, adsorption, molecular rearrangement, and physical cross-linking. These processes are modulated by the structural proper-ties of the surfactants, system conditions, and the presence of active petroleum components such as asphaltenes and resins [8,26,53]. Thus, understanding the formation of viscoelastic interfacial films during surfactant injection requires considering both surfactant properties and their interactions with native crude oil components.

Surface activity plays a central role in film formation, as it determines the adsorption ability of surfactants and asphaltenes. Depending on their relative affinities, adsorption may be competitive or cooperative, leading to more viscous or more elastic interfaces [54,55]. Tail length, electrostatic attraction, and aromatic or hydrogen-bonding functionalities also contribute to the formation of tightly packed films with gel-like structures at the interface [26,53].

### 5.1. Adsorption Kinetics of Surfactants at Interfaces

**Figure 6:**
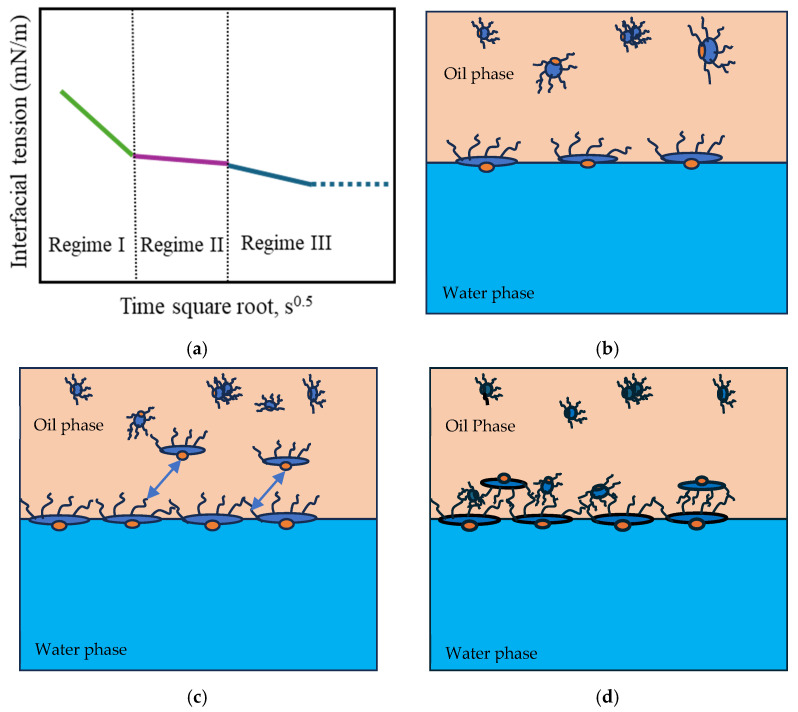
(**a**) Typical dynamic interfacial tension response of asphaltene in toluene solution adsorbing at the oil/water interface. (**b**) Schematic of the adsorption of planar asphaltenes at the interface in Regime I. (**c**) Schematic of the adsorption of planar asphaltenes at the interface in Regime II, when steric hindrance started to slow down the adsorption. The arrows symbolize the steric effect. (**d**) Schematic of asphaltene adsorption at the interface in Regime III. Adapted from Zhang [57].

The adsorption of amphiphilic molecules at the oil–water interface occurs through distinct regimes, governed by diffusion, steric hindrance, and molecular rearrangement [56,57]. A typical dynamic IFT profile reflects three main regimes, as illustrated in Figure 6.

In Regime I, the initial interfacial tension (IFT) decay rate follows a linear relationship with the square root of time (Equation (7)). During this Regime, the most significant reduction in IFT occurs as a large number of surfactant molecules diffuse from regions of higher concentration (the bulk phase) to regions of lower concentration (the interface). This diffusion-driven mechanism allows the molecules to adsorb onto the available interfacial area due to their surface activity (Figure 6b). The concentration of surfactants and asphaltenes at the interface (Γ, number of molecules per m^2^) is governed by the bulk concentration (C, number of molecules per m^3^), the diffusion coefficient (D), and time (t), following the relationship described in Equation (7) [58].

(7) $$\Gamma =2C\sqrt{\frac{D}{\pi }t}$$

The adsorption time, at constant bulk and interface concentration of surfactant or asphaltenes, depends on the diffusion coefficient (D) related to the hydrodynamic radius (R), the system absolute temperature (*T*), the Boltzmann constant ($K_{B}$), and continuous phase viscosity (η) according to the Stokes–Einstein equation [59]), as given by:

(8) $$D=\frac{K_{B}T}{6\pi \eta R}$$

The diffusion coefficient of asphaltene ranges between 2.2 × 10^−10^ m^2^/s and 8.2 × 10^−14^ m^2^/s [58,60,61,62], and depends on the ability of aggregation of asphaltenes, whereas surfactants are reported between 1.2 × 10^−9^ and 2.5 × 10^−11^ m^2^/s, depending on surfactant concentration and its state to form cluster and micelles [62,63,64,65]. According to these molecular diffusion studies, several authors have reported that the duration of Regime I is short, typically ranging from 0.33 to 20 min for asphaltenes and surfactants [26,66].

Similarly, the number of surfactant or asphaltene molecules permanently adsorbed at the interface depends on the surface activity of each molecule, which is directly related to the compositional and structural properties of the surfactants, as well as the system conditions, such as temperature, salinity, and viscosity [67,68].

In Regime II, the diffusion of additional molecules is hindered by steric effects once the excess surface concentration reaches a critical value [57]. At this point, molecules largely occupy most interfacial sites, and the newly arriving molecules require additional energy to be adsorbed onto the remaining available sites [69]. As a result, the reduction in interfacial tension slows significantly, and the IFT–time curve approaches a plateau, as shown in Figure 6a,c [57,59,70].

**Figure 7:**
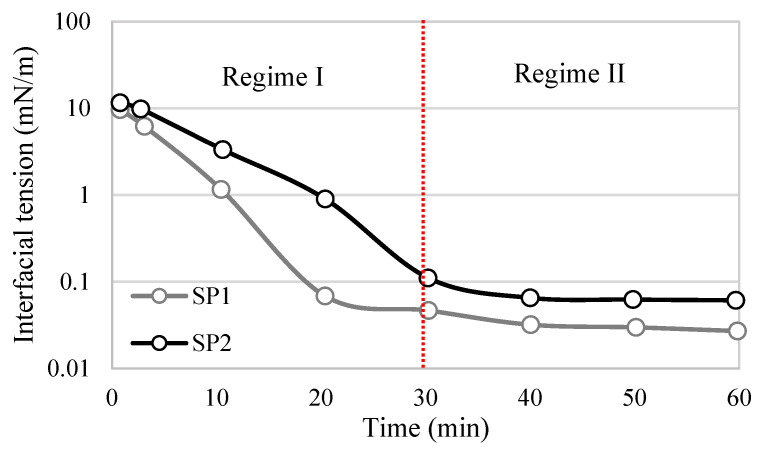
Behavior of interfacial tension in time through two regimes for surfactant-polymeric (SP) with heavy oil. Adapted from Cao et al. [7].

Regime III differs for asphaltenes and surfactants due to differences in their interfacial structures. In the case of asphaltenes, the rate of interfacial tension further decreases, and the continuous adsorption of asphaltenes occurs mainly through adsorption into the sublayer of the interface, as well as the reconfiguration of adsorbed molecules and aggregations via van der Waals interactions [59] (Figure 6d). This is the longest stage during which additional adsorption and molecular reconfiguration occur due to cross-linking of the molecular chains. These processes result in a long-term decrease in the dynamic IFT and contribute to the formation of viscoelastic films, depending on the intramolecular interactions [26]. Regime III concludes when a balance between interactions is achieved, and the interfacial tension reaches a plateau. Regime III is commonly negligible for surfactant films, as interactions between molecular segments required to form a sublayer are unlikely to occur. Consequently, no further reduction in interfacial tension is observed (Figure 7). However, during this regime, surfactants can still undergo intramolecular interactions, forming a more elastic interface through hydrogen bonding and π-π interactions.

### 5.2. Modification of Interfacial Properties in Regime III

During Regime III, surface-active molecules already adsorbed at the oil–water interface interact either competitively or cooperatively, leading to the formation of viscous, elastic, or viscoelastic films. The structural features of surfactants play a decisive role in determining whether these films remain fluid, evolve into gel-like layers, or achieve a balance between viscosity and elasticity. The following subsections discuss the influence of specific molecular properties on interfacial organization.

#### 5.2.1. Surfactant Tail Length

The hydrocarbon chain length is a critical parameter influencing surfactant hydrophobicity and adsorption behavior. Longer tails decrease the critical micelle concentration (CMC) while increasing excess surface concentration, thereby lowering interfacial tension at a fixed bulk concentration [67]. Stronger hydrophobic interactions between extended chains promote denser molecular packing, provided that steric constraints of the headgroups are compatible [71].

**Figure 8:**
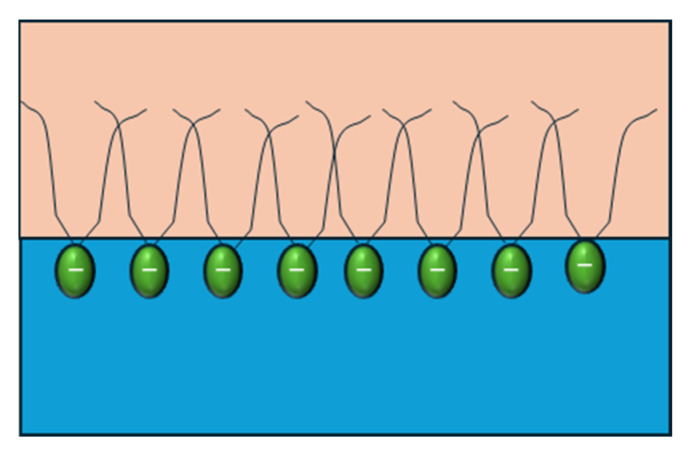
Graphical representation of the length of the surfactant’s tails. Adapted from Zhang et al. [55].

In an experimental study, Y. Zhang et al. [55] demonstrated that increasing the alkyl chain length of surfactant molecules enhances hydrophobic interactions, driving the formation of more compact and cohesive interfacial films at the oil–water interface (Figure 8). This denser molecular packing strengthens intermolecular cohesion within the adsorption layer, leading to significantly higher dilatational elasticity. Conversely, surfactants with shorter alkyl chains form weaker, less organized films that are more prone to molecular exchange with the bulk phase.

Similar outcomes were reported by Dong et al. [72], who found that extending the hydrocarbon chain in surfactants consistently increased film compactness and interfacial elasticity. These results highlight the critical role of chain length in reinforcing interfacial viscoelasticity, as longer hydrophobic tails promote more stable and elastic films that can resist interfacial deformation [72].

#### 5.2.2. Effect of Branching or Unsaturation

Branching or unsaturation within the hydrophobic chain modifies surfactant solubility and packing ability at the interface. Branched or unsaturated chains typically enhance solubility relative to straight-chain isomers, altering the balance between interfacial activity and bulk aggregation. Importantly, surfactants with double chains show markedly higher interfacial activity than single-chain analogs, forming tightly packed films through enhanced tail–tail interactions [55]. Jin et al. [73] reported that grafted branched chains increased intermolecular interactions at the oil–water interface, producing rigid interfacial films. Such structures can stabilize emulsions and suppress oil snap-off, which may improve residual oil mobilization but simultaneously complicate emulsion separation in production facilities [73].

#### 5.2.3. Interactions in Surfactants Mixtures

**Figure 9:**
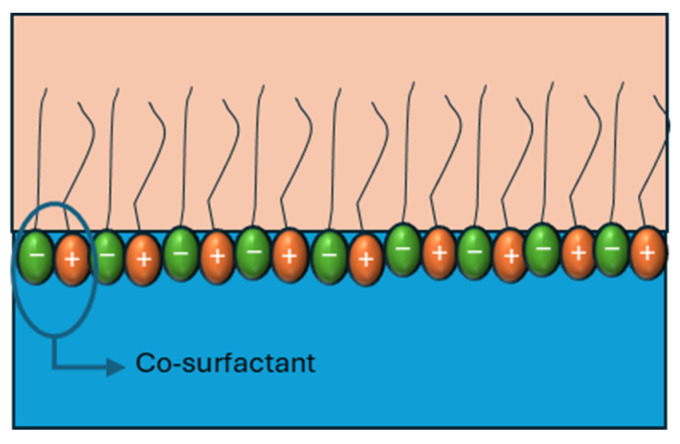
Graphical representation of the interaction of surfactant mixture effects on interfacial viscoelasticity. Adapted from Wang et al. [53] and Han et al. [74].

When cationic and anionic surfactants are combined, the difference between the charges of the head group induces electrostatic interactions, promoting the formation of closer-packing interfaces (Figure 9).

When cationic and anionic surfactants are combined, the electrostatic attraction between oppositely charged headgroups promotes closer molecular packing at the interface, thereby enhancing interfacial elasticity (Figure 9). Wang et al. [53] demonstrated that mixtures of anionic surfactants (SDS, SDDS, and DAS) with the cationic surfactant C12TAB in a 1:1 ratio exhibited elasticity values that correlated directly with the headgroup charge of the anionic species. Their results showed that the electrostatic attraction followed the order SDS > SDDS > DAS, and the dilatational elasticity modulus increased in the same sequence (Table 3), confirming the direct relationship between electrostatic strength and interfacial reinforcement.

Similar synergistic effects have been reported in other systems. Chen et al. [75] observed that mixtures of C12-Gly-Na and C12TAB displayed obvious electrostatic synergy, significantly enhancing interfacial elasticity compared to the individual surfactants [75]. Han et al. (2023) [74] further expanded on this mechanism, showing that cationic/anionic surfactant systems can associate into so-called “pseudo-co-surfactants,” where two molecules act cooperatively as if they possessed two hydrophobic tails. This association greatly strengthens the mixed interfacial layer and improves its stability [74].

## 6. Competitive and Cooperative Interactions of Surfactants

In multicomponent systems, the behavior of the oil–water interface depends strongly on whether surface-active species interact competitively or cooperatively. These mechanisms determine whether interfacial films become loose and viscous or compact and elastic, with direct implications for emulsion stability and enhanced oil recovery.

***Competitive interactions***. When multiple amphiphilic molecules coexist, the one with the highest interfacial activity preferentially adsorbs at the oil–water boundary, displacing fewer active species. Such competitive adsorption often leads to an interface dominated by a single component, resulting in a loosely packed film with lower viscoelastic moduli and reduced interfacial tension.

Such mechanisms are particularly relevant in petroleum systems, where native asphaltenes act as natural surfactants and compete with injected surfactants for adsorption sites. If asphaltenes exhibit higher interfacial activity, they generate rigid interfacial films that increase interfacial tension [76]. Conversely, when surfactants have greater activity, they can displace asphaltenes, forming more fluid interfaces with lower interfacial tension. This competitive mechanism is central to emulsion destabilization, where demulsifying surfactants disrupt rigid asphaltene films by replacing them at the interface [30,67,77].

Competition is also evident in mixed surfactant systems. Zhang et al. [55] showed that when two surfactants with significantly different surface activities are combined, the more active surfactant (SNN10) displaces the less active one (SB12). As a result, the viscoelasticity and interfacial tension of the mixture are indistinguishable from those observed with SNN10 alone. These findings emphasize that in systems with large differences in surface activity, cooperative effects are suppressed, and competitive adsorption dictates interfacial properties [55].

***Cooperative interactions.*** In contrast, when amphiphiles exhibit comparable interfacial activities, they can co-adsorb and interact cooperatively to form more elastic interfacial films. Cooperative interactions are enhanced when surfactant headgroups or functional moieties can form favorable electrostatic or hydrogen-bonding interactions. For example, when asphaltenes and surfactants have similar surface activity, co-adsorption leads to denser interfaces and moderate IFT values. Such conditions are highly favorable for surfactant injection in EOR, as they enable the simultaneous achievement of high elastic moduli and low interfacial tension, both of which are critical for mobilizing trapped oil [7,11].

A clear example of this mechanism was provided by Jiang et al. [78], who studied the interaction between asphaltenes and an anionic surfactant (dodecyldiglycol) [78]. Their results showed that, individually, surfactant and asphaltenes produced only modest reduction in IFT, with surface activities differing by less than 0.4 mN/m. Under these conditions, co-adsorption occurred, with aromatic groups of asphaltenes aligning parallel to the interface, while surfactant headgroups and oxygenated asphaltene moieties oriented perpendicularly. This cooperative arrangement produced a dense interfacial film, further reducing IFT and stabilizing the oil–water interface.

## 7. Interfacial Interactions Between Asphaltenes and Surfactants

The interaction between surfactants and asphaltenes at the oil–water interface depends strongly on molecular structure and composition. One of the most significant intermolecular interactions is π–π stacking, arising from electronic interactions between aromatic rings. This effect, widely reported in asphaltenes and in aromatic surfactants, is a major contributor to the formation of viscoelastic films [79,80,81].

**Figure 10:**
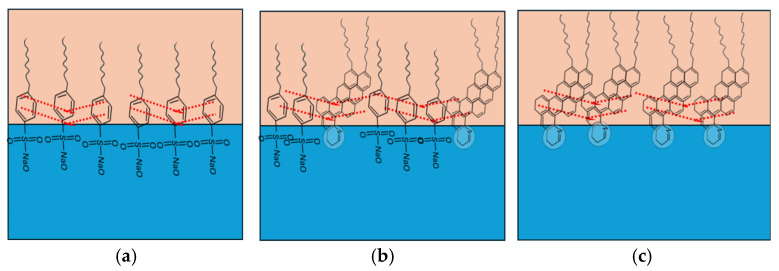
Intermolecular reaction between (**a**) SDBS molecule, (**b**) active asphaltenes and SDBS, and (**c**) active material of asphaltenes. The dotted red lines represent the π–π stacking.

As illustrated in Figure 10a, sodium dodecylbenzene sulfonate (SDBS) arranges at the oil–water interface with its benzene rings oriented in the hydrophobic tails, facilitating π–π stacking (dotted red lines) [49,81]. These interactions enhance interfacial rigidity and elasticity by forming compact and uniform films with low IFT and negative interfacial potential. At surfactant concentrations above the CMC, SDBS molecules are tightly arranged, creating compressed interfacial films where π–π stacking dominates, leading to highly stable emulsions [81].

In contrast, the presence of asphaltenes introduces additional complexity. Active asphaltenes (~2 wt% of total) are primarily responsible for rigid, thick films that stabilize the interface, though they are difficult to displace due to strong steric repulsions [59,82]. Non-active asphaltenes, however, form softer films that can partially desorb under compression–relaxation or be replaced by surfactants with high surface activity. Structural studies indicate that asphaltenes tend to adopt a “face-to-face” stacking configuration (Figure 10c), where aromatic cores align parallel to the interface, aliphatic chains extend into the oil phase, and polar groups anchor into the water. This arrangement promotes π–π stacking, hydrogen bonding, and porous elastic networks [70,83,84].

When SDBS is introduced into asphaltene-containing systems, cooperative interactions occur. SDBS molecules penetrate the asphaltene interfacial layer into the oil phase, forming cross-linked structures through π–π stacking and van der Waals interactions between polycyclic aromatic hydrocarbons and the surfactant tails (Figure 10b). Hydrogen bonding further stabilizes these cross-linked films, creating dense elastic interfacial structures [7,26,49].

In contrast to the cooperative stabilization between SDBS and asphaltenes, surfactants lacking aromatic rings, such as sodium dodecyl sulfate (SDS), can destabilize asphaltene films. Studies have shown that SDS reduces π–π interactions, causing asphaltenes to be drawn from the interface into the bulk oil phase [85]. Ahmadi & Chen (2020) [86] demonstrated that SDS was the most effective dispersant compared to nonionic (TX-100), cationic (CTAB), and amphoteric (CAPB) surfactants. This is due to the strong anionic headgroup of SDS (–O–SO_3_^−^), which contains four hydrogen bond acceptors, whereas CTAB offers only one, and TX-100 and CAPB form weaker hydrogen bonds [86].

Finally, surfactants such as polyoxyethylene nonylphenol (Igepal CO series) exhibit distinctive interfacial effects. Fan et al. [26] reported that the addition of Igepal to asphaltene-containing systems converted the interface from highly elastic to predominantly viscous. This behavior was attributed to the rapid adsorption of Igepal at the interface, which displaced and dispersed asphaltenes into the bulk phase, thereby disrupting π–π stacking and hydrogen-bonding interactions among asphaltene molecules [26].

## 8. Impact of Surfactant Concentration on Interfacial Properties

Both surfactant composition and concentration are critical factors in determining interfacial activity, as they influence intermolecular interactions and the dynamic exchange between bulk and interface [53,63]. Depending on concentration, interfacial behavior can evolve towards greater elasticity or viscosity, reflecting changes in the strength and relaxation of the interfacial film.

The impact of surfactant concentration on interfacial properties is determined by a balance between adsorption at the interface, molecular exchange with the bulk, and specific intermolecular interactions. Stubenrauch [87] provided a general framework to explain why dilatational elasticity typically exhibits a maximum with increasing concentration. At low concentrations, adsorption dominates, followed by an increase in surface coverage, and intermolecular interactions are strengthened, thereby enhancing elasticity [87]. At high concentrations, however, the molecular exchange rate between the bulk and the interface accelerates, even out interfacial tension gradients and reduces elasticity. The crossover between these two opposing processes manifests as a maximum in the elasticity–concentration curve. The exact position of this maximum depends on the static and dynamic adsorption parameters of each surfactant, which explains why different systems show maxima at different concentrations [88,89,90]. Table 4 summarizes how surfactant type and molecular structure influence interfacial rheology.

This classical single-maximum trend is well illustrated by conventional ionic surfactants such as SDS, C12TAB, and SDBS [53,91,92]. In these systems, elasticity increases with concentration to a peak, then gradually decreases to near zero as exchange dominates, forming predominantly viscous interfacial films. However, more complex behaviors have been reported for surfactants with additional intermolecular interactions. Amino acid surfactants such as C12-Gly-Na exhibit a second maximum at high surface coverage, arising from hydrogen bonding between amide groups that rigidify the interfacial film [75].

Similarly, nonionic surfactants containing polyoxyethylene groups (EO) (e.g., TX100, TX165, TX405) and Gemini surfactants with EO spacers display two maxima in dilatational elasticity, as EO chains act as flexible, hydrated segments that compress and expand under deformation. At higher concentrations, EO–EO and EO–water associations further reinforce the interfacial film [67,72]. These peculiarities in rheological response become more pronounced as the ethylene oxide chain length increases. When a sufficient number of EO units are present, the spacer can penetrate the aqueous phase and adopt a loop-like conformation, allowing hydrophilic spacers to interconnect and form a structured interfacial sublayer [93].

In addition, the incorporation of propylene oxide (PO) groups in extended surfactants produces a distinctive effect, as PO units increase the interfacial dilatational modulus by hindering the dynamic relaxation of the interfacial film, slowing molecular rearrangement and orientation [33]. Consequently, EO/PO-extended surfactants can form interfacial films with controllable viscoelasticity, typically exhibiting phase angles between 10° and 50°, even under ultra-low interfacial tension conditions (0.2–2 mN/m). This unique combination of elasticity and low IFT is of particular importance for enhancing oil displacement efficiency in EOR [33].

In some cases, the exchange mechanism is almost fully suppressed. For double-chain surfactants such as SNN10 (an anionic surfactant with a sulfonate headgroup and two decyl tails), Y. Zhang et al. [55] reported a continuous increase in elasticity with concentration, without any observable maximum, achieving an angle phase between 10° and 20° at an interfacial tension of roughly 0.34 mN/m [55]. Similar monotonic behavior has been observed for surfactants with longer hydrophobic tails [72] and in systems with strong electrostatic interactions between headgroups [53]. In such cases, the interfacial film becomes so densely packed that molecular exchange is negligible, leaving adsorption and intermolecular interactions as the dominant mechanisms.

In summary, the classical framework of Stubenrauch [87] which involves a crossover between adsorption-driven reinforcement and exchange-driven relaxation, provides a useful baseline for understanding concentration effects [87]. However, subsequent studies demonstrate that surfactant molecular architecture (headgroup chemistry, chain number and length, presence of aromatic or EO groups) and environmental conditions (ionic strength) can significantly modify this balance. Depending on these factors, surfactants may exhibit a classical single maximum, dual maxima driven by specific interactions, or a monotonic increase in elasticity when exchange is fully suppressed.

## 9. Future Research Directions and Prospects

This review highlights the central role of interfacial rheology in determining the stability and dynamics of surfactant–asphaltene films at oil–water interfaces. Evidence shows that surfactant concentration, molecular architecture, and intermolecular interactions such as hydrogen bonding and π–π stacking critically modulate interfacial viscoelasticity. Conventional ionic surfactants often display the classical single-maximum behavior of dilatational elasticity [53,87] while more complex architectures (e.g., amino acid, Gemini, or EO/PO-extended surfactants) can induce dual maxima or continuous increases in elasticity, depending on the suppression of molecular exchange with the bulk [33,72]. Similarly, competitive and cooperative interactions between surfactants and asphaltenes determine whether interfaces become looser and more viscous or more elastic and resistant to deformation [7,11]

Despite this progress, several critical knowledge gaps remain. Most notably, the direct correlation between interfacial rheological properties and oil recovery efficiency remains underexplored in surfactant injection. Current evidence suggests that interfaces with moderate elasticity and low phase angles are more effective in suppressing snap-off and promoting oil mobilization. However, this relationship has not been systematically validated in surfactant-EOR studies. Based on current insights, we hypothesize that EO/PO-extended surfactants, when combined with sufficiently long hydrophobic tails and headgroups capable of strong lateral interactions (electrostatic attraction, hydrogen bonding, or π–π stacking), can form viscoelastic films with phase angles between 10° and 50° even at ultra-low interfacial tensions (0.1–1 mN/m). These conditions are highly desirable for controlling snap-off phenomena and increasing the oil recovery factor beyond what can be achieved by ultra-low IFT and viscous films alone [11,22]. Furthermore, the inclusion of aromatic groups in surfactant structures may reinforce this effect through π–π stacking, thereby promoting cooperative interactions with asphaltenes while maintaining low interfacial tension.

To test these hypotheses, research should focus on bridging molecular-scale interfacial rheology with pore-scale recovery dynamics, combining advanced rheological measurements with microfluidic visualization and core-flooding experiments under reservoir-representative conditions. We specifically recommend evaluating how systems with different viscoelastic properties influence oil droplet size and detachment during snap-off, how elasticity affects emulsion stability, and how these combined effects translate into oil displacement efficiency. Another promising direction is the systematic study of extended surfactants containing EO/PO chains, examining the impact of chain number, tail length, and the presence of aromatic groups in the hydrophobic structure on viscoelastic proper-ties and, ultimately, on oil recovery performance. These systems can be evaluated in the presence and absence of asphaltenes to corroborate the effect of competitive or cooperative interactions.

Advancing the field of interfacial rheology in surfactant–asphaltene systems will re-quire an interdisciplinary approach that combines experimental innovation, molecular modeling, and field validation. Addressing these research directions will bring the scientific community closer to developing predictive, sustainable, and efficient surfactant formulations for enhanced oil recovery while also ensuring manageable emulsion stability in production operations.

## 10. Summary and Conclusions

This review summarizes current knowledge on the interfacial rheology of oil–water interfaces formed by active petroleum components and surfactants commonly employed in EOR and emulsification processes. Surfactants enhance oil recovery by reducing interfacial tension (IFT) and capillary forces, aiding in oil displacement and improving fluid flow. The formation of viscoelastic films at the oil–water interface is crucial for stabilizing emulsions and controlling the breakup of oil droplets, thereby reducing residual oil saturation. Achieving the right balance between IFT and viscoelasticity is essential, since excessive elasticity can hinder oil recovery by preventing oil droplet coalescence. Although interfacial rheology provides valuable insights for optimizing surfactant formulations, its direct application to crude oil emulsions and recovery efficiency remains limited, underscoring the need for further studies.

In oil–water systems, surfactants and asphaltenes may either compete or cooperate at the interface. In competitive interactions, molecules with higher interfacial activity displace others, forming weaker films with reduced viscoelasticity. In contrast, cooperative interactions between surfactants and asphaltenes with comparable interfacial activity produce denser and more elastic films, lowering IFT while enhancing emulsion stability and oil recovery. The most favorable outcomes for EOR occur when such synergistic effects generate interfacial layers that simultaneously achieve low IFT and desirable viscoelastic properties.

The type of interaction depends strongly on molecular structure and concentration. For instance, aromatic surfactants such as SDBS engage in π–π stacking with asphaltenes, thereby reinforcing the rigidity of interfacial films. In contrast, SDS disrupts these associations by dispersing asphaltenes into the bulk. Similarly, active asphaltenes contribute to the formation of strong elastic interfaces, whereas non-active fractions form softer, less stable films. These findings highlight the importance of tailoring surfactant structure, headgroup chemistry, tail length, and presence of aromatic or EO/PO groups to modulate interfacial properties.

Both surfactant and asphaltene concentrations also play a decisive role. Increasing surfactant concentration reduces IFT through enhanced adsorption, but excessive molecular exchange at high concentrations can weaken elasticity. Likewise, higher asphaltene concentrations promote aggregation and cross-linking, leading to more stable interfacial films. Ultimately, interfacial behavior arises from a complex balance between adsorption, molecular exchange, and intermolecular interactions.

Overall, the reviewed evidence underscores the intricate interplay between surfactants, asphaltenes, and interfacial rheology in oil–water systems. Future progress will require optimizing these interactions to achieve surfactant formulations that not only improve oil displacement efficiency in porous media but also ensure controllable emulsion stability during production.

## Figures and Tables

**Table 1:** A comparison of main methods used to measure interfacial rheology.

Method	Frequency Range (Hz)	Advantages	Limitations	Ref
Bicone rheometer	0.01–10	- Cost-effective and straightforward setup. - Suitable for stiff interfaces and systems with high interfacial viscosity.	- Requires a large sample volume. - Complex alignment and setup. - Not suitable for fragile interfaces with very low viscosity.	[17,26,31,32]
Ring geometry (Du Noüy or double-wall ring)	0.01–10	- Lightweight design with low moment of inertia. - Enables steady and oscillatory shear measurements. - Applicable to viscous and viscoelastic interfaces.	- Fragile geometry; limited durability. - Low sensitivity for small moduli. - Bulk contributions from submerged parts.	[15,16,17,19,32]
Pendant drop	0.001–1	- Broad frequency range. - Simple setup and easy operation. - Requires small sample volumes. - Suitable for dynamic interfacial behavior studies.	- Limited sensitivity in ultra-low IFT systems. - Applicable only at low frequencies.	[27,28]
Spinning drop	0.001–1	- Ideal for ultra-low interfacial tension systems. - Requires small sample volumes. - Straightforward and reproducible operation.	- Artifacts from surfactant convection or viscous bulk losses. - Possible wall–drop interactions. - Limited frequency range.	[12,32,33,34]

**Table 2:** A comparison of results relating the phase angle to the oil recovery factor with double wall ring rheometer published by [11].

Crude Oil Sample Name	Phase Angle $\left(\phi \right)$	Oil Recovery Factor (%)	Interfacial Tension (mN/m)
WG	16	43	5
TC	26	81	10
RC	45	45	17

**Table 3:** Relation between head group charge of mixtures and elastic modules [53].

Surfactants	Head Group Charge	Mixtures	Elastic Modules (mN/m)
SDS	−1.33	SDS:C12TAB	42
SDDS	−1.00	SDS:C12TAB	16.5
DAS	−0.91	SDS:C12TAB	5.0
C12TAB	0.59		

**Table 4:** A summary of the type and molecular structure effect on interfacial rheology.

Surfactant (Type)	Effect on Interfacial Rheology	Ref
SDBS (Anionic, aromatic)	Aromatic rings enable π–π stacking at the oil–water interface, forming compact, elastic films with low IFT. In asphaltene systems, SDBS promotes cross-linked networks stabilized by π–π and hydrogen bonding, resulting in highly rigid and stable interfaces.	[7,26,49]
SDS (Anionic, aliphatic)	Without aromatic groups, SDS interactions are dominated by electrostatic repulsion and exchange with the bulk phase. Elasticity rises to a single maximum with concentration, then decreases as molecular exchange dominates.	[53,85,91,92]
CTAB/C12TAB (Cationic)	Adsorbs strongly onto negatively charged interfaces via electrostatic attraction. Elasticity increases to a peak, followed by viscous-dominated behavior at high surface coverage.	[53,85,86]
Cationic–Anionic Mixtures	Electrostatic attraction between oppositely charged headgroups enhances interfacial packing and elasticity, producing stable, densely packed films with limited molecular exchange.	[53,74,75]
Nonionic EO-based (TX100, TX165, TX405)	Hydrated EO chains act as flexible segments that compress and expand under deformation, producing two elasticity maxima. Long EO chains promote loop conformations and the formation of a structured interfacial sublayer.	[63,67,68,89,93]
EO/PO-Extended Surfactants	PO groups slow interfacial relaxation and molecular reorientation, increasing the dilatational modulus. These systems show tunable viscoelasticity (phase angle 10–50°) even under ultra-low IFT.	[33,67,72]
Double-Chain Surfactants	Strong hydrophobic interactions suppress molecular exchange, leading to a continuous increase in elasticity with concentration and highly rigid films.	[55,72]
Long-Chain Surfactants	Longer hydrophobic tails enhance van der Waals interactions and interfacial packing, leading to monotonic increases in elasticity and the formation of dense, stable films.	[53,72]

## Data Availability

No new data were created or analyzed in this study. Data sharing is not applicable to this article.
